# Small airway remodeling in acute respiratory distress syndrome: a study in autopsy lung tissue

**DOI:** 10.1186/cc9401

**Published:** 2011-01-06

**Authors:** Maina MB Morales, Ruy C Pires-Neto, Nicole Inforsato, Tatiana Lanças, Luiz FF da Silva, Paulo HN Saldiva, Thais Mauad, Carlos RR Carvalho, Marcelo BP Amato, Marisa Dolhnikoff

**Affiliations:** 1 Department of Pathology, Experimental Air Pollution Laboratory-LIM05, Sao Paulo University Medical School, Av Dr. Arnaldo, 455, São Paulo, 01246-903, Brazil; 2 Pulmonary Division, Heart Institute (InCor), Sao Paulo University Medical School, Av Dr Enéas Carvalho de Aguiar, 44, São Paulo, 05403-904, Brazil

## Abstract

**Introduction:**

Airway dysfunction in patients with the Acute Respiratory Distress Syndrome (ARDS) is evidenced by expiratory flow limitation and dynamic hyperinflation. These functional alterations have been attributed to closure/obstruction of small airways. Airway morphological changes have been reported in experimental models of acute lung injury, characterized by epithelial necrosis and denudation in distal airways. To date, however, no study has focused on the morphological airway changes in lungs from human subjects with ARDS. The aim of this study is to evaluate structural and inflammatory changes in distal airways in ARDS patients.

**Methods:**

We retrospectively studied autopsy lung tissue from subjects who died with ARDS and from control subjects who died of non pulmonary causes. Using image analysis, we quantified the extension of epithelial changes (normal, abnormal and denudated epithelium expressed as percentages of the total epithelium length), bronchiolar inflammation, airway wall thickness, and extracellular matrix (ECM) protein content in distal airways. The Student's *t*-test or the Mann-Whitney test was used to compare data between the ARDS and control groups. Bonferroni adjustments were used for multiple tests. The association between morphological and clinical data was analyzed by Pearson rank test.

**Results:**

Thirty-one ARDS patients (A: PaO_2_/FiO_2 _≤200, 45 ± 14 years, 16 males) and 11 controls (C: 52 ± 16 years, 7 males) were included in the study. ARDS airways showed a shorter extension of normal epithelium (A:32.9 ± 27.2%, C:76.7 ± 32.7%, *P *< 0.001), a larger extension of epithelium denudation (A:52.6 ± 35.2%, C:21.8 ± 32.1%, *P *< 0.01), increased airway inflammation (A:1(3), C:0(1), *P *= 0.03), higher airway wall thickness (A:138.7 ± 54.3 μm, C:86.4 ± 33.3 μm, *P *< 0.01), and higher airway content of collagen I, fibronectin, versican and matrix metalloproteinase-9 (MMP-9) compared to controls (*P *≤0.03). The extension of normal epithelium showed a positive correlation with PaO_2_/FiO_2 _(r^2 ^= 0.34; *P *= 0.02) and a negative correlation with plateau pressure (r^2 ^= 0.27; *P *= 0.04). The extension of denuded epithelium showed a negative correlation with PaO_2_/FiO_2 _(r^2 ^= 0.27; *P *= 0.04).

**Conclusions:**

Structural changes in small airways of patients with ARDS were characterized by epithelial denudation, inflammation and airway wall thickening with ECM remodeling. These changes are likely to contribute to functional airway changes in patients with ARDS.

## Introduction

Acute Respiratory Distress Syndrome (ARDS) is characterized by inflammation-mediated alveolar/capillary barrier dysfunction with interstitial and airspace protein-rich edema fluid, resulting in ventilation-perfusion mismatch and consequent severe hypoxemia [1]. Several ventilatory strategies are implemented in these patients to restore adequate oxygenation; however, mechanical ventilation itself can increase damage to the lung tissue [2]. The inflammatory changes, the loss of airspace capacity secondary to lung collapse and the dynamic reopening of distal lung units during mechanical ventilation, result in a marked decrease in lung compliance. Furthermore, an increase in lung resistance has also been reported, which was partially attributed to impaired peripheral airway function [3]. Studies that report expiratory flow limitation and dynamic hyperinflation in patients with ARDS also indicate that these functional alterations are related to airway closure [4-7]. Recent studies suggest a role for distal airway epithelium injury in the pathophysiology of human acute lung injury (ALI) and propose that Clara cell CC16 protein levels in plasma and pulmonary edema fluid can be used as a biomarker for the diagnosis of ALI/ARDS [8].

Several experimental models have been proposed to reproduce the functional and morphological lung changes in ARDS. Models of ventilation-induced lung injury have shown that ventilation of normal or lavaged lungs with low end-expiratory lung volume causes a persistent increase in airway resistance and histological evidence of peripheral airway injury characterized by bronchiolar epithelial necrosis and sloughing and rupture of alveolar-bronchiolar attachments [9-13]. These morphological and functional alterations have been mainly attributed to shear stresses caused by cyclic opening and closing of peripheral airways [3,7].

Since airway mechanics is largely dependent on airway structure, extracellular matrix (ECM) composition and distribution, in addition to airway-parenchyma interdependence forces, the functional airway alterations observed in ARDS patients are likely associated with airway morphological changes [14]. Although both human and experimental studies have suggested that airway changes contribute to impaired lung function in acute lung injury, no study to date has focused on distal airway morphological changes in the lungs of human subjects with ARDS. Therefore, the aim of the present study was to analyze the structural and inflammatory changes in small airways of patients with ARDS. For this purpose, we measured the extent of epithelial alterations, airway dimensions and the expression of major lung ECM elements and their regulators within the small airway walls of patients with ARDS submitted to autopsy and compared them with control subjects. We further correlated the airway changes to clinical data and mechanical ventilation parameters.

## Materials and methods

This is a retrospective study using archived material from routine autopsies performed at the Autopsy Service of Sao Paulo University Medical School. The study was approved by the institutional review board for human studies (CAPPesq-FMUSP). Consent for performing autopsy was obtained from the next of kin of all the subjects involved in the study.

### Study population

Thirty-one patients with ARDS submitted to autopsy between 2004 and 2007 were retrospectively included in the study. Inclusion criteria were clinical diagnosis of ARDS [15], histological findings of diffuse alveolar damage [16], an absence of chronic lung diseases, and sufficient archived autopsy material (at least three small airways per patient) for analyses. ARDS was defined as the 1994 American-European Consensus criteria [15]_, _that is, acute onset, the ratio of arterial oxygen tension to the fraction of inspired oxygen (PaO_2_/FiO_2_) ≤200, bilateral infiltrates on chest radiograph and no left atrial hypertension. Twenty-three non-smoker patients who died of non-pulmonary causes, without previous lung diseases were selected for controls. From these, 12 were excluded due to histological lung alterations (bronchopneumonia, pulmonary edema, and pulmonary hemorrhage) and 11 patients with normal lung histology were used as controls. The following clinical data were assessed in medical charts: age, gender, predisposing cause of ARDS, days of ARDS evolution (time interval between ARDS diagnosis and death), and values of PaO_2_, plateau pressure, positive end-expiratory pressure (PEEP), driving pressure and PaO_2_/FiO_2_.

### Tissue processing

Paraffin blocks of lung tissue collected during autopsy were retrieved from the archives of the Department of Pathology of Sao Paulo University Medical School. In the routine autopsies, three to four fragments of lung tissue were collected from any regions of altered lung parenchyma. In normal lungs, one fragment of lung tissue was collected from each lobe. The tissue had been previously fixed in 10% buffered formalin for 24 hours, routinely processed and paraffin embedded. Five μm-thick sections were stained with hematoxylin and eosin (H&E) and with Weigert's Resorcin-Fuchsin staining for elastic fibers [17]. The following proteins were identified with immunohistochemistry (IHC) as previously described [18]: collagens type I (COLI) and type III (COLIII), fibronectin, versican and matrix metalloproteinases (MMP) -2 and -9. Antibody types and pre-treatments used are shown in Table 1.

**Table 1:** Antibodies and processing used in immunohistochemical analyses

Antibody	Pre-treatment	Specie	Clone	Dilution	Origin
Collagen I	Citrate	Goat	Polyclonal	1:1000	US Biological-Swampscott, Massachusetts/USA
Collagen III	Trypsin	Mouse	III-53	1:100	Oncogene & Calbiochem, Darmstadt/Germany
Fibronectin	Citrate	Rabbit	Polyclonal	1:6000	Dako, Glostrup/Denmark
Versican	Trypsin	Mouse	2-B-1	1:100	Seikagaku CO, Tokyo/Japan
MMP-2	Citrate	Mouse	A-Gel VC2	1:3000	LabVision, Fremont/USA
MMP-9	Citrate	Mouse	56-2A4	1:50	Calbiochem, Darmstadt/Germany

MMP, matrix metalloproteinase.

### Morphological analysis

**Figure 1:**
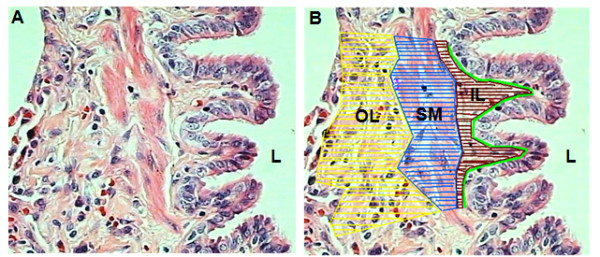
**A schematic representation of image analysis**. **(A) **Small airway stained with H&E. **(B) **The same airway subdivided into epithelium (EP), inner layer (IL), smooth muscle (SM), and outer layer (OL). L = lumen. The green line represents the basement membrane.

Only transversely cut small airways were analyzed, defined as those showing a short/long diameter ratio greater than 0.6 [19]. Small airways were defined based on their epithelial basement membrane (BM) perimeter (BM perimeter ≤6 mm) [19,20]. Airways were subdivided into epithelial layer, inner layer (located between the epithelium and the internal smooth muscle border), smooth muscle (SM), and outer layer (located between the external SM border and the alveolar parenchyma) (Figure 1) [19]. In each airway, the entire circumference was analyzed at a 400× magnification by an investigator blinded to the study group. Measurements were taken using image analysis with the software Image-Pro^® ^Plus 4.1 for Windows^® ^(Media Cybernetics-Silver Spring, MD, USA) on a personal computer connected to a digital camera coupled to a microscope (Leica DMR, Leica Microsystems Wetzlar GmbH, Wetzlar, Germany).

The following parameters were analyzed on H&E stained slides: 1) the extent of normal epithelium, abnormal epithelium (epithelial cells with histological signs of necrosis and/or degeneration) and denudated epithelium (BM exposed) expressed as a percent of the total epithelium length; 2) thickness of the inner, SM and outer airway layers and the total airway wall expressed as layer area corrected by the corresponding BM perimeter (μm^2^/μm); and 3) inflammation index determined semiquantitatively based on the presence of inflammatory cells infiltrating the airway wall, using a four-grade scale: absent = 0, minimal inflammation = 1, moderate inflammation = 2 and marked inflammation = 3 [13].

The content of ECM proteins and MMPs was determined in IHC-stained slides. The area of ECM proteins positive staining was determined in three airway regions: inner, SM and outer layers. The area of MMP-positive staining was determined in two airway regions: epithelial layer and total airway wall (including the inner and outer layers and SM together). Protein content was expressed as positive area divided by airway BM length (μm^2^/μm).

### Clinical-morphological correlations

Since lung injury can be observed after a few hours of mechanical ventilation [12], we investigated the possible association of morphological airway changes with mechanical ventilation and disease severity in ARDS patients who died within 48 hours after the diagnosis (*n *= 16). For this purpose we correlated the morphological parameters with ventilatory parameters (mean values of plateau pressure, driving pressure and PEEP) and PaO_2_/FiO_2 _value obtained at the moment of the clinical diagnosis.

### Statistical analysis

Data are presented as mean ± SD or median (interquartile range). After testing for distribution of data, the Student's *t*-test or the Mann-Whitney test were used to compare data between the ARDS and control groups. Bonferroni adjustments were used for multiple tests. The association between morphological and clinical data was analyzed by Pearson rank test. Statistical analysis was performed using the statistical package SPSS 15.0 (SPSS, Chicago, IL, USA). The level of significance was set at *P *< 0.05.

## Results

### Study population

Demographic and clinical data of ARDS patients (*n *= 31) and controls (*n *= 11) are presented in Table 2. The mean ± SD age of ARDS patients and controls was 45 ± 14 and 52 ± 16 years, respectively (*P *= 0.18). The main predisposing factors for ARDS were pneumonia (42%) and sepsis (35%). Pulmonary ARDS (ARDSp) and extrapulmonary ARDS (ARDSext) accounted for 52% (*n *= 16) and 48% (*n *= 15) of the patients, respectively. These two subgroups were also similar with respect to age (ARDSp = 45.9 ± 13.6 years and ARDSext = 43.6 ± 4.3) and gender (ARDSp = 7 F/9 M and ARDSext = 8 F/7 M). Days of ARDS evolution (time interval between ARDS diagnosis and death) ranged from 1 to 24; however, most patients (71%) died in the first week, and 52%, within the first 48 hours (Table 3). Ventilatory parameters and PaO_2_/FiO_2 _values of ARDS patients are presented in Table 3. Median values of plateau pressure, PEEP and driving pressure were 28(10) cmH_2_O, 11(8) cmH_2_O and 15(5) cmH_2_O, respectively. All patients were ventilated using a lung-protective strategy with a low tidal volume (≤6 mL/Kg). Control patients did not receive mechanical ventilation. One control subject died at home, seven controls arrived at the emergency room in cardiorespiratory arrest and three controls were admitted to the hospital for non-pulmonary conditions. All control patients were submitted to resuscitation maneuvers for a maximum of one hour. All control patients were non-smokers.

**Table 2:** Clinical data of Acute Respiratory Distress Syndrome (ARDS) patients and controls

Characteristics	ARDS	Controls
Number	31	11
Age (years) ^α^	44 (20)	48 (25)
Gender (M/F)	16/15	7/4
**Predisposing factor for ARDS, n (%)**		NA
Pneumonia	13 (42)	
Sepsis	11 (35)	
Aspiration	2 (6)	
Pancreatitis	2 (6)	
Hypovolemic shock	2 (6)	
Alveolar bleeding	1 (3)	
**Co-morbidities, n (%)**		
Systemic arterial hypertension	9 (20)	6 (54)
Chronic hepatopathy	9 (20)	1 (9)
AIDS	8 (17)	--
Diabetes mellitus	5 (11)	3 (27)
Systemic Lupus Erythematosus	3 (6.5)	--
Pneumocystosis	3 (6.5)	--
Pulmonary hypertension	1 (2.2)	--
Multiple sclerosis	1 (2.2)	--
Multiple myeloma	1 (2.2)	--
Crohn disease	1 (2.2)	--
Chagas disease	1 (2.2)	1 (9)
Schistosomosis	1 (2.2)	--
Acute myeloid leukemia	1 (2.2)	--
Lymphoma	1 (2.2)	--
Tuberculosis	1 (2.2)	--
Epilepsy	--	1 (9)
**Primary Cause of Death, n (%)**		
Multiorgan failure	14 (45)	
Refractory sepsis	7 (23)	
Respiratory failure	4 (13)	
Thoracic bleeding	2 (6)	
Gastrointestinal bleeding	4 (13)	1 (9)
Cardiovascular diseases		9 (82)
Vesical bleeding		1 (9)
Total length hospitalization, days	15 (19)	0 (0.08)

Data are given as median (interquartile range) or n (percentage).

M, masculine; F, feminine; AIDS, acquired Immunodeficiency Syndrome; NA, non applicable.

**Table 3:** Days of evolution, ventilatory parameters and PaO_2_/FiO_2 _values of Acute Respiratory Distress Syndrome (ARDS) patients

Patient	Days of ARDS	**Plateau pressure **^**α**^	**PEEP **^**α**^	**Driving pressure **^α^	**PaO**_**2**_**/FiO**_**2**_
1	1	18	08	10	161.0
2	1	35	20	15	93.2
3	1	12	06	06	170.7
4	1	28	14	12	82.0
5	1	21	9	12	93.7
6	1	25	10	15	68.1
7	1	30	08	22	79.5
8	1	40	10	30	47.2
9	1	40	15	25	51.0
10	2	25	10	15	166.3
11	2	32	22	10	137.3
12	2	35	17	18	104.2
13	2	28	13	15	133.5
14	2	23	08	15	130.8
15	2	27	14	13	156.5
16	2	21	11	10	111.7
17	3	38	18	20	84.0
18	3	30	14	16	166.2
19	4	25	11	14	197.0
20	6	21	09	12	149.7
21	6	25	10	15	196.7
22	7	21	07	14	189.7
23	8	28	11	17	126.3
24	9	29	17	12	119.7
25	10	30	14	16	108.3
26	13	22	09	13	153.2
27	14	28	17	11	195.6
28	16	20	10	10	186.7
29	19	39	18	21	199.0
30	22	25	14	11	182.4
31	24	37	20	17	173.7

Median (IQR)	2 (8)	28 (10)	11 (8)	15 (5)	137 (80)

PaO_2_/FiO_2, _ratio of arterial oxygen tension to the fraction of inspired oxygen.

PEEP, positive end-expiratory pressure.

For each patient, plateau pressure and PEEP correspond to mean values in the first 48 hours.

PaO_2_/FiO_2 _correspond to values assessed at the time of clinical diagnosis.

^α ^cmH_2_O.

### Morphological analysis

All available transversely cut small airways for each patient were analyzed, varying from three to six airways per patient (mean of 3.6 per patient in each staining). A total of 1,218 airways were analyzed considering all the stainings used. The median perimeter of small airways for ARDS patients and controls was 1.94(1.13) mm and 2.06(1.21) mm, respectively, corresponding to small membranous bronchioles [21]. There was no significant difference in airway perimeter between ARDS patients and controls.

**Figure 2:**
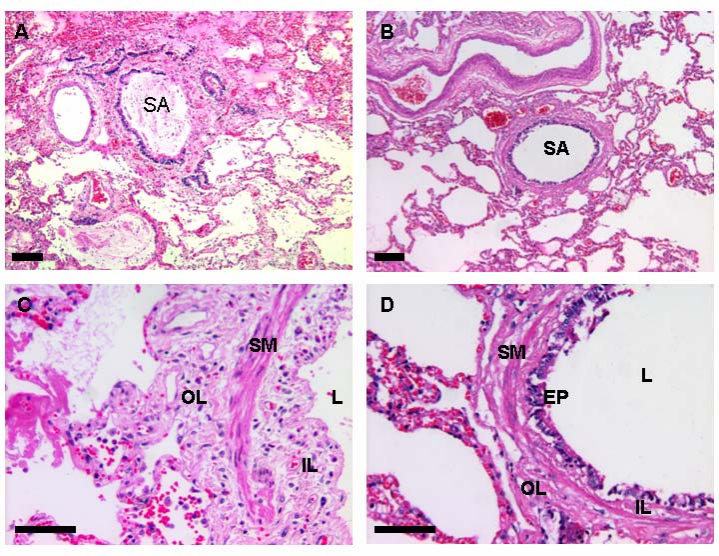
**Lung histology from ARDS and control patients**. Representative photomicrographs of distal airway and alveolar tissue from ARDS **(A **and **C) **and control **(B **and **D) **patients. ARDS lungs show extensive intra-alveolar exudate (A) and small airway thickening with mild inflammation and epithelium denudation (C). SA = airway; L = lumen; EP = epithelium; SM = smooth muscle; OL = outer layer; IL = inner layer. H&E staining. Scale bars: A and B = 100 μm, C and D = 50 μm.

Structural and inflammatory data are presented in Table 4 and Figure 2. There was a significantly lower extent of normal epithelium (*P *< 0.001) and a higher extent of abnormal epithelium (*P *= 0.007) and epithelial denudation (*P *= 0.015) in the ARDS group compared to controls. The ARDS group showed a significantly higher thickness of the total airway wall, inner layer and outer layer compared to controls (*P *≤0.03). The inflammation index was also higher in ARDS patients than in controls (*P *= 0.03).

**Table 4:** Structural and inflammatory data on small airways in ARDS patients and controls

	ARDS	Controls	*P*
Normal epithelium	32.9 ± 27.2	76.7 ± 32.7	< 0.001
Abnormal epithelium	14.4 ± 14.8	1.37 ± 3.20	0.007
Denudated epithelium	52.6 ± 35.2	21.8 ± 32.1	0.015
Airway wall thickness	138.7 ± 54.3	86.4 ± 33.3	0.005
Inner layer thickness	35.2 ± 32.0	17.2 ± 8.14	0.034
Smooth Muscle thickness	14.8 ± 8.20	18.5 ± 22.7	0.427
Outer layer thickness	88.7 ± 29.9	50.4 ± 17.7	< 0.001
Inflammation index [median(range)]	1(3)	0(1)	0.027

ARDS, acute respiratory distress syndrome.

Values are expressed as mean ± SD, unless otherwise specified. Epithelium parameters are expressed as a percentage of the airway basement membrane perimeter (%). Airway layers are presented as area (μm^2^) corrected by the basement membrane perimeter (μm).

**Figure 3:**
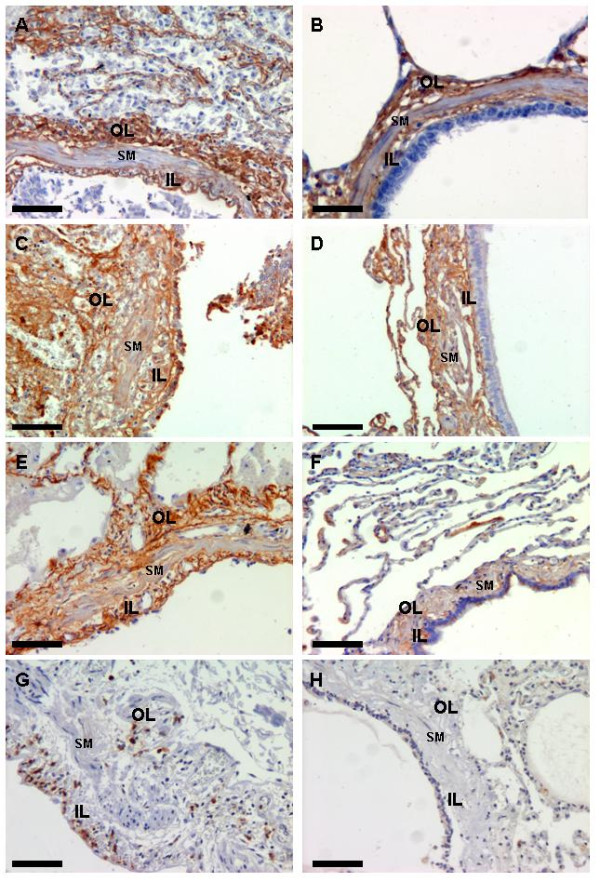
**Representative photomicrographs of extracellular matrix proteins and matrix metalloproteinase-9 expression in the airways**. Photomicrographs of small airways from ARDS patients **(A, C, E, G) **and controls **(B, D, F, H) **stained with anti-collagen I (A and B), anti-fibronectin (C and D), anti-versican (E and F) and anti-MMP-9 (G and H). ARDS airways show higher content of collagen I, fibronectin and versican and higher MMP-9 expression by inflammatory cells. L = lumen; SM = smooth muscle; OL = outer layer; IL = inner layer. Scale bars = 50 μm.

**Figure 4:**
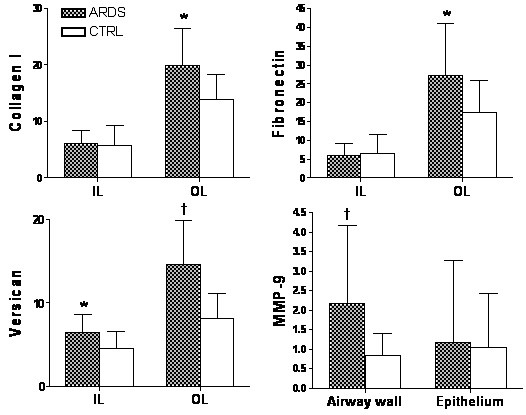
**Protein content in ARDS patients and controls**. The graphs show collagen I, fibronectin, versican and MMP-9 content (μm^2^/μm) in the small airways of ARDS patients and controls. IL = inner layer, OL = outer layer. Bars represent mean; lines represent SD. *P ≤0.03 compared with controls; †*P *< 0.003 compared with controls.

Immunoreactivity of ECM components and MMPs showed similar patterns of distribution in the lung tissue of both ARDS patients and controls. MMPs showed positive staining mainly in inflammatory cells (mostly monocytes/macrophages and PMNs) and weak expression in airway epithelial cells and SM cells. Representative photomicrographs of airway ECM and MMP-9 expression are shown in Figure 3. Figure 4 shows protein content in ARDS patients and controls. We observed higher content of COL I, fibronectin and versican in the outer airway layer of ARDS patients compared to controls (*P *≤0.03). Versican expression was also higher in the inner airway layer in ARDS patients (*P *< 0.02). There were no differences in COL III and elastic fiber content between the two groups, and no difference in the content of ECM proteins within the SM layer. We observed increased expression of MMP-9 in the ARDS group only in the airway wall (*P *= 0.003), with no differences observed within the epithelial layer. There were no differences in MMP-2 expression between the two groups.

Comparisons between ARDSp and ARDSext subgroups showed higher levels of MMP-9 in the airways from the ARDSp group (*P *= 0.03). There were no significant differences in the inflammation index or in any structural parameter between the two subgroups. To evaluate the airway inflammation and structural changes over the course of the disease, we also categorized our ARDS patients into two subgroups according to time interval between ARDS diagnosis and death, as follows: Ards1 = 1 to 6 days (21 patients) and Ards2 = ≥7 days (11 patients). We did not find any significant differences in inflammatory or structural parameters between the two subgroups.

### Clinical-morphological correlations

**Figure 5:**
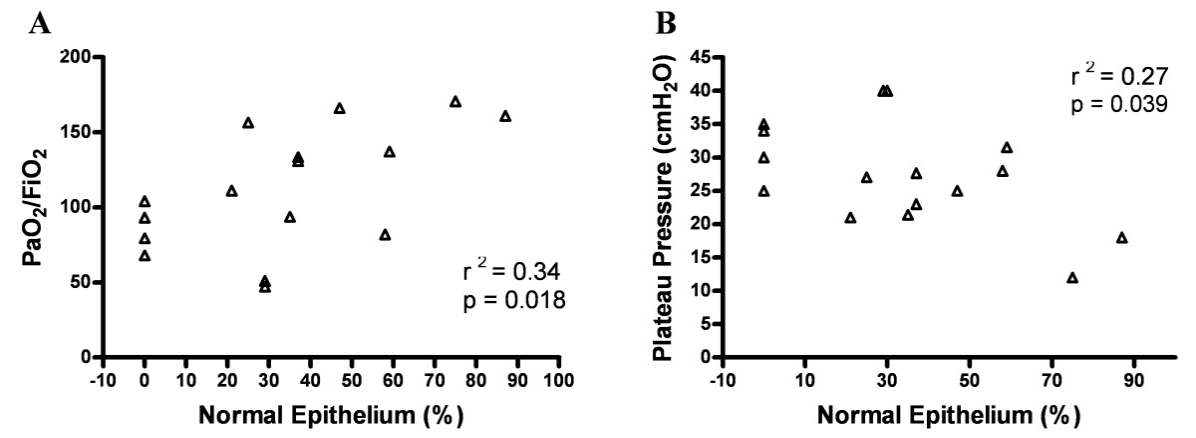
**Clinical-morphological correlations in ARDS patients**. The graphs show the correlation (Pearson test) between epithelial morphological changes and PaO_2_/FiO_2 _**(A) **and plateau pressure **(B) **in patients who died within 48 hours after the clinical diagnosis (*n *= 16).

The extension of normal epithelium showed a significant positive correlation with PaO_2_/FiO_2 _(r^2 ^= 0.34; *P *= 0.018) and a negative correlation with plateau pressure (r^2 ^= 0.27; *P *= 0.039) (Figure 5). In addition, the extension of denuded epithelium showed a negative correlation with PaO_2_/FiO_2 _(r^2 ^= 0.27; *P *= 0.038). There was no correlation between values of PEEP or driving pressure and the morphological parameters.

## Discussion

In the present study, we analyzed for the first time the structural changes in small airways in patients with ARDS compared to control subjects. Our main findings were the presence of epithelial denudation, airway inflammation and increased thickness of small airway walls with deposition of collagen I, fibronectin and versican, mainly localized to the outer wall.

Recent studies have suggested that the peripheral airways play an important role in the pathophysiology of ALI/ARDS [3]; however, no study has focused on airway morphological changes in lungs from human subjects with ARDS. Experimental models of ALI are used to investigate these changes and have shown epithelial necrosis and denudation in distal airways of animals ventilated with low lung volumes [9-12]. Our results are in line with these experimental studies and show that bronchiolar epithelium denudation is also present in humans and is associated with ARDS severity. The mechanisms of epithelial injury and denudation are not completely understood but are likely to result from changes in shear stress due to reopening of either collapsed small airways or non-collapsed flooded airways [7,22] and from stretch-induced hyperdistension of epithelial cells [23]. Gadigliali and Gaver (2008) suggested that this increased stress on the airway epithelial cell lining may induce significant cellular deformations, cell death, and/or disruption of cell adhesions. The damaged epithelial cell may in turn upregulate inflammatory pathways and/or alter surfactant secretion [24].

Airway inflammation is diffusely present in surfactant-depleted lungs of rabbits submitted to low end-expiratory lung volume ventilation [13] and in rats submitted to high volume-induced lung injury [23]. Our results show that distal airway inflammation is also present in human ARDS lungs. Whether airway inflammation in ARDS represents a response of terminal bronchioles to the primary insult or is rather a spreading of inflammatory cells from the alveolar tissue is not clear. In either case we suggest that airway inflammation is likely to be involved in the pathogenesis of airway remodeling in ARDS.

Previous studies evaluating ECM changes in ARDS have shown altered alveolar septa with lung fibrosis [25] and increased alveolar content of collagen and elastic fibers [26-28], fibronectin [29] and versican [30] in exudative and/or proliferative phases of lung injury. The fibroproliferative process characterized by collagen deposition, even in the early phase of ARDS, is associated with a severe reduction in respiratory system compliance [31]. We show for the first time that remodeling is also present at the terminal bronchiolar level and suggest that these structural airway changes may also have functional implications. The functional consequences of airway remodeling are dependent on which layer in the airway wall is changed as well as on the composition and mechanical properties of the material that is altered [32]. The inner area provides resistance of the tissue to compression; the smooth muscle layer is usually altered in pulmonary diseases characterized by bronchoconstriction; and the outer wall is directly attached to the lung parenchyma and is, therefore, crucial for the maintenance of lung tissue structure and transmission of elastic forces. Thus, we believe that airway compartmentalization provides important insight toward a better understanding of structure-function relationships in pulmonary diseases. Airway dysfunction in patients with ARDS who are ventilated with low PEEP is characterized by expiratory flow limitation and gas trapping [4,5], which have been related to airway closure and inhomogeneous distribution of ventilation [6,7]. The mechanisms involved in airway closure include surfactant dysfunction and a decrease in airway-parenchyma interdependence secondary to interstitial edema, alveolar collapse, and possibly the rupture of alveolar attachments [3,11,12]. Since the outer airway layer is the main region where mechanical forces are transmitted from the alveolar parenchyma to the airway wall, it represents a critical site that may be affected by both the inflammatory process and the mechanical damage caused by abnormal stress. Interestingly, in this study collagen I, fibronectin and versican levels were primarily increased in the outer airway wall, which could contribute to airway-parenchyma uncoupling by altering the mechanical interdependence between these two compartments.

The higher MMP-9 expression seen in the ARDS group is in accordance with previous studies showing increased levels of MMP-9 in the bronchoalveolar lavage of patients with ARDS [33,34]. The observation of increased MMP-9 expression in inflammatory cells within the airway wall suggests that MMP-9 may be involved in airway ECM remodeling in ARDS patients. Increased MMP-9 expression could either be associated with higher ECM turnover within the airway wall or represent a response to excessive matrix deposition in an attempt to restore equilibrium to the ECM composition.

In chronic airway inflammatory lung diseases, airway remodeling is correlated with marked changes in airway mechanics and symptoms related to airway obstruction [14]. Although airway obstruction is not a characteristic of ALI, patients who survive ARDS can present mild to moderate abnormalities in lung function evidenced by decreased FEV_1_, FVC and/or FEV_1_/FVC evaluated one to three years after hospital discharge [35-37]. It is possible that the persistence of these pulmonary function changes is related to airway remodeling.

To determine if the airway changes were different in patients with distinct predisposing factors, ARDS patients were divided into pulmonary and extrapulmonary subgroups. Although we observed higher levels of MMP-9 in the airways from the ARDSp subgroup, there were no differences in the inflammation index or in any structural parameter between the ARDSp and ARDSext subgroups. These findings suggest that the airway alterations in ARDS were the result of the inflammatory insult (and/or ventilator injury), independent of the primary cause. We also categorized our ARDS patients into two subgroups according to time interval between ARDS diagnosis and death. We did not find any significant differences in inflammatory or structural parameters between patients who died in the first week and patients who died more than seven days after diagnosis, suggesting that the airway alterations were present at the start of the syndrome and were maintained over time.

Pulmonary injury is heterogeneously distributed in ARDS, resulting in inhomogeneous ventilation and predisposing the lung to ventilator-induced lung injury [2]. Previous studies suggest that lung injury in ALI is more severe in the atelectatic dependent lung regions [38,39]; however, more recent studies have suggested that the peripheral airway injury observed in experimental ALI is diffusely distributed in both dependent and non-dependent regions [13]. One limitation of our study was the retrospective analysis of lung tissue, which did not allow us to systematically assess regional differences in airway injury in these lungs from human subjects with ARDS. Due to the retrospective character of the study, another limitation was the lack of systematic recording of clinical data. In many charts, information regarding smoking habits or the specifics of the lung mechanics was not available, which could have influenced the interpretation of our results. Furthermore, since we only analyzed tissue from patients who died, the extent to which the results obtained in the present study can be transposed to the less severe cases of ARDS is unclear.

Although the observed airway changes are likely to play a role in the pathogenic mechanisms of ALI, it is not clear if these changes are due to the insult leading to ARDS or to ventilator injury. It is well known that pulmonary injury in ARDS patients can be exacerbated by the ventilatory strategy [2], as indicated by clinical trials showing significantly higher mortality among patients who received ventilation with high tidal volume and high inspiratory plateau pressures [40-42]. In our patients, airway epithelial injury showed significant correlations with PaO_2_/FiO_2 _and inspiratory pressure values, suggesting that both the primary pulmonary insult leading to ARDS and the ventilator injury are associated with airway structural alterations in ARDS patients. Rouby *et al. *[43] analyzed the histological aspects of pulmonary barotrauma in critically ill patients with acute respiratory failure and observed in 6 out of 30 lungs severe damage to terminal bronchioles characterized by bronchiolar dilation, epithelial hyperplasia and metaplasia. Similarly to our results, the authors suggested that mechanical ventilation with a high peak airway pressure plays a role in the pathogenesis of bronchial injury and airspace enlargement.

## Conclusions

Our results revealed structural changes in the small airways of patients with ARDS, characterized by epithelial denudation, inflammation and airway wall thickening with ECM remodeling. These small airway alterations are likely to contribute to impaired lung function in patients with ARDS.

## Key messages

• Patients with ARDS show evidence of airway dysfunction characterized by expiratory flow limitation and dynamic hyperinflation. These functional alterations have been attributed to closure/obstruction of small airways. Airway morphological changes have been reported in experimental models of acute lung injury, characterized by epithelial necrosis and denudation in distal airways.

• In the present study, we analyzed for the first time the structural changes in distal airways in ARDS patients, which were characterized by epithelial denudation, inflammation and airway wall thickening with extracellular matrix remodeling.

• These small airway alterations are likely to contribute to impaired lung function in patients with ARDS.

## Abbreviations

ALI: acute lung injury; ARDS: acute respiratory distress syndrome; BM: basement membrane; COLI: collagen type I; COLIII: collagen type III; ECM: extracellular matrix; H&E: hematoxylin and eosin; IHC: immunohistochemistry; MMP: matrix metalloproteinases; PaO_2_/FiO_2_: ratio of arterial oxygen tension to the fraction of inspired oxygen; PEEP: positive end-expiratory pressure; SM: smooth muscle.

## Competing interests

The authors declare that they have no competing interests.

## Authors' contributions

MMBM participated in the design of the study, carried out the immunohistochemistry reactions and the morphometric analyses, performed the statistical analysis and drafted the manuscript. RCPN and NI helped to carry out the morphometric analyses and were involved in drafting the manuscript. TL helped to carry out the morphometric analyses, was involved in the acquisition of clinical data and was involved in revising the manuscript. LFFS participated in the design of the study, performed the histological analysis and the immunohistochemistry quality control, helped at the statistical analysis and was involved in drafting the manuscript. PHNS and TM contributed to the conception and design of the study and were involved in drafting the manuscript. CRRC and MBPA contributed to the conception and design of the study, contributed to analysis and interpretation of data and were involved in revising the manuscript. MD conceived the study, performed the histological analysis, performed the immunohistochemistry quality control, performed the statistical analysis, drafted and revised the manuscript. All authors read and approved the final version of the manuscript.
